# Age-Dependent Clinical Patterns of Primary Epstein–Barr Virus Infection in Children: Insights for Diagnostic Accuracy

**DOI:** 10.3390/pathogens15050554

**Published:** 2026-05-20

**Authors:** Demet Teker-Düztaş, Ayşe Kaman, Gönül Tanır

**Affiliations:** 1 Departments of Pediatrics, Dr. Sami Ulus Children’s Health and Diseases Training and Research Hospital, 06080 Ankara, Türkiye; 2 Departments of Pediatric Infectious Disease, Dr. Sami Ulus Children’s Health and Diseases Training and Research Hospital, 06080 Ankara, Türkiye; ayse092003@yahoo.com (A.K.);

**Keywords:** Epstein–Barr virus infection, infectious mononucleosis, pediatric patients, age-related clinical features, antibiotic stewardship, public health implications

## Abstract

Primary Epstein–Barr virus (EBV) infection in children exhibits substantial clinical heterogeneity, often complicating early diagnosis and leading to unnecessary antibiotic use. This retrospective study evaluated 695 children (0–18 years) diagnosed with primary EBV infection at a tertiary pediatric center between 2010 and 2015, defined by positive viral capsid antigen (VCA) IgM and negative Epstein–Barr nuclear antigen (EBNA) IgG. Clinical, laboratory, and ultrasonographic findings were compared according to age group (≤4 vs. >4 years) and clinical setting (inpatient vs. outpatient). The median age was 3.75 years (IQR: 2–6.25), and more than half of the patients were ≤4 years. Younger children more frequently presented with nonspecific respiratory and gastrointestinal symptoms, whereas older children more commonly exhibited the classic infectious mononucleosis (IM) phenotype, including sore throat, dysphagia, lymphadenopathy, and hepatosplenomegaly (*p* < 0.001). Antibiotics were prescribed in 64.2% of patients, while 21.7% required hospitalization. Multivariable logistic regression analyses demonstrated that age was not an independent predictor of hospitalization, classic IM phenotype, or antibiotic use. Instead, specific clinical and laboratory findings—such as lymphopenia, lymphadenopathy, vomiting, thrombocytosis, and tonsillar hypertrophy—emerged as the key determinants of clinical outcomes. To enhance diagnostic discrimination, receiver operating characteristic (ROC) analysis of ANC/ALC and AST/ALT ratios was performed, and a composite risk score (0–2) was derived. Although both markers showed modest discriminative ability (AUC 0.607 and 0.575), their high negative predictive values (>90%) suggest potential utility as rule-out tools. The composite score demonstrated a stepwise increase in the probability of classic IM presentation across age groups. In conclusion, primary EBV infection demonstrates a clear age-related clinical spectrum; however, clinical and laboratory features rather than age alone drive key outcomes. These findings highlight the need for age-specific diagnostic strategies and improved antimicrobial stewardship, while the proposed risk score provides a foundation for future validation studies.

## 1. Introduction

Epstein–Barr virus (EBV) is a double-stranded DNA virus belonging to the *Herpesviridae* family and represents one of the most common viral pathogens in childhood. The clinical spectrum of EBV infection ranges from asymptomatic cases to life-threatening complications. This variability in clinical presentation is largely attributed to the host immune response, particularly lymphocytic infiltration and cytokine release following viral transmission [1,2,3]. The intensity of the immune response depends on the host’s age, immune status, environmental hygiene, and socioeconomic conditions [4]. From an epidemiological perspective, primary EBV infection in developing countries typically occurs during early childhood and is most often asymptomatic or presents with mild symptoms [5]. In contrast, in developed countries, the infection tends to occur at older ages and commonly manifests in adolescents and young adults as infectious mononucleosis (IM), characterized by fever, lymphadenopathy, and tonsillopharyngitis, accompanied by lymphocytosis and atypical lymphocytes in peripheral blood [6,7,8]. Children aged four years and younger are reported to be more susceptible to EBV infection [9], and the clinical presentation tends to be atypical in younger children but manifests with the classic mononucleosis features in older ones [10].

Comprehensive characterization of the clinical and laboratory features of EBV infection in children is essential for accurate diagnosis and optimal management. Nevertheless, data on age-specific variations in disease course and outcomes are scarce, with most available studies focusing primarily on hospitalized cases [9,10,11,12,13]. These gaps emphasize the need for comprehensive investigations of primary EBV infections in pediatric outpatients and for studies that better reflect real-world clinical practice. The aim of this study was to evaluate the demographic, clinical, laboratory, and prognostic characteristics of children diagnosed with primary Epstein–Barr virus (EBV) infection, with a particular focus on age-related clinical variations and differences between hospitalized and outpatient cases. In addition to these primary objectives, we also aimed to identify independent predictors of key clinical outcomes and to evaluate the diagnostic utility of selected laboratory markers for the classic IM phenotype.

## 2. Materials and Methods

### 2.1. Study Population

This study included patients aged 0–18 years who presented to Dr. Sami Ulus Maternity and Children’s Training and Research Hospital between January 2010 and December 2015 and were diagnosed with primary Epstein–Barr virus (EBV) infection. Medical records were retrospectively reviewed using the hospital’s electronic database. Primary EBV infection was defined serologically as positivity for EBV viral capsid antigen (VCA) IgM and Epstein–Barr nuclear antigen (EBNA) IgG negativity [2]. Patients whose medical records were unavailable or who tested positive for EBNA IgG were excluded. Participants were categorized into two age groups (≤4 years and >4 years), and comparisons were also made between outpatients and hospitalized patients. The study was conducted in accordance with the Declaration of Helsinki and approved by the Institutional Review Board of Dr. Sami Ulus Maternity and Children’s Training and Research Hospital (Approval No: 73799008-1464).

### 2.2. Demographic and Clinical Characteristics

Patients’ presenting symptoms—including fever, sore throat, cervical swelling, dysphagia, rhinorrhea, cough, poor appetite, vomiting, diarrhea, rash, and eyelid edema—along with physical examination findings such as tonsillar hypertrophy, fever, lymphadenopathy, tonsillitis, rash, periorbital edema, hepatomegaly, and splenomegaly were recorded. Patients presenting with the classic IM triad—fever, pharyngeal swelling, and sore throat—were analyzed separately [6]. Symptoms, presence of the IM triad, and physical findings were compared between age groups and between inpatients and outpatients.

### 2.3. Laboratory and Imaging Evaluation

At admission, laboratory data—including complete blood count [Hemoglobin (Hgb), white blood cell (WBC), platelet count (PLT), absolute neutrophil count (ANC), absolute lymphocyte count (ALC)], liver transaminases [aspartate aminotransferase (AST) and alanine aminotransferase (ALT)], erythrocyte sedimentation rate (ESR), C-reactive protein (CRP), and the presence of Downey cells on peripheral smear—were recorded and analyzed by age group and follow-up type. Throat culture results were evaluated when available. Rapid *Streptococcus A* antigen testing was not routinely performed during the study period; therefore, corresponding data were not systematically available. EBV serology (VCA IgM, EBNA IgG) was tested by ELISA using a Triturus analyzer (Grifols, Barcelona, Spain) and Vircell^®^ kits (Vircell, Granada, Spain) according to the manufacturers’ instructions. In patients with cervical lymphadenopathy detected by ultrasonography, the location of the lymph nodes was documented, and abdominal ultrasonography was performed to assess hepatomegaly and/or splenomegaly. Findings were compared across age groups and between inpatient and outpatient cohorts. Data on hospitalization, antibiotic use, length of hospital stay, and recovery time were also collected. Clinical recovery was defined as the resolution of symptoms—such as fever, sore throat, and fatigue—along with normalization of hepatosplenomegaly and lymphadenopathy on physical examination [14].

### 2.4. Statistical Analysis

All statistical analyses were performed using IBM SPSS Statistics for Windows, version 22.0 (IBM Corp., Armonk, NY, USA). The normality of continuous variables was assessed using the Kolmogorov–Smirnov test in conjunction with visual inspection of histograms. Normally distributed variables were expressed as mean ± standard deviation (SD), whereas non-normally distributed variables were presented as median with interquartile range (IQR; 25th–75th percentile). Categorical variables were summarized as frequencies and percentages. Associations between categorical variables were evaluated using the chi-square test or Fisher’s exact test, as appropriate. Yates’ continuity correction was applied for 2 × 2 contingency tables when necessary. Comparisons of continuous variables between two groups were performed using Student’s *t*-test or Mann–Whitney U test, depending on data distribution.

To identify independent predictors of key clinical outcomes, three multivariable binary logistic regression models were constructed with hospitalization status, classic infectious mononucleosis (IM) phenotype, and antibiotic use as dependent variables. Independent variables included age group, presenting symptoms, physical examination findings, and selected laboratory parameters (WBC, CRP, ANC/ALC ratio, AST/ALT ratio, and group A ß-hemolytic *Streptococcus* [GABHS] positivity). Model fit was evaluated using the likelihood ratio chi-square test and McFadden’s pseudo-R^2^. Multicollinearity was assessed prior to model construction. Results are reported as odds ratios (ORs) with 95% confidence intervals (CIs).

The diagnostic performance of ANC/ALC and AST/ALT ratios in identifying the classic IM phenotype was evaluated using receiver operating characteristic (ROC) curve analysis. Optimal cut-off values were determined using the Youden index, and corresponding sensitivity, specificity, positive predictive value (PPV), and negative predictive value (NPV) were calculated. A composite risk score (range 0–2) was derived by assigning one point for each ratio at or below. The association between the composite score and the classic IM phenotype was assessed using multivariable logistic regression adjusted for age group. Estimated marginal probabilities were calculated across combinations of risk score categories and age groups. Discriminative performance was expressed as the area under the curve (AUC) with 95% confidence intervals. *p*-value < 0.05 was considered statistically significant.

## 3. Results

### 3.1. Demographic Data

During the study period, a total of 852 patients were identified as EBV VCA IgM-positive. Of these, 128 patients with EBNA IgG positivity and 29 patients with unavailable records were excluded, leaving 695 eligible participants, 401 of whom (57.7%) were male. The median age was 3.75 years (IQR: 2–6.25 years). Among them, 372 (53.5%) were aged ≤4 years, 323 (46.5%) were >4 years, and 44 (6%) were adolescents (>10 years). Although more cases were observed in October and November, the overall monthly distribution did not differ significantly (*p* = 0.444), suggesting no clear seasonal pattern.

### 3.2. Clinical Presentation and Age-Based Differences in Symptoms

Analysis of presenting complaints showed that the most frequent symptoms were fever (60.6%, *n* = 421), sore throat (48.3%, *n* = 336), and cervical swelling (38.1%, *n* = 265) (Table 1). IM triad was identified in 93 patients (13.3%) and was significantly more common in children older than four years (*p* < 0.001). When symptoms were compared by age group, rash (*p* < 0.001), diarrhea (*p* < 0.001), cough (*p* < 0.001), and rhinorrhea (*p* = 0.002) were more frequent among children aged ≤4 years, whereas sore throat (*p* < 0.001), dysphagia (*p* = 0.002), and poor appetite (*p* = 0.022) were more common in those >4 years (Table 1).

### 3.3. Physical Examination Findings

On physical examination, the most common findings were tonsillar hypertrophy (66%, *n* = 459), lymphadenopathy (52.4%, *n* = 364), and tonsillitis (38.8%, *n* = 270) (Table 1). Among patients with lymphadenopathy, 59.3% had submandibular, 40.4% had cervical, and 0.3% had supraclavicular node involvement. Comparison of physical findings by age group showed that rash was more frequent in children aged ≤4 years (*p* < 0.001), whereas lymphadenopathy (*p* < 0.001) and hepatosplenomegaly (*p* = 0.002) were more common in those >4 years. Rash occurred following antibiotic use in 100 patients (51.2%), and antibiotic-associated rash was significantly more frequent among children aged ≤4 years (*p* < 0.001) (Table 1).

### 3.4. Laboratory and Imaging Findings

Complete blood count results were available for 675 patients (97.1%). When compared by age, children aged ≤4 years had a lower median hemoglobin level (*p* < 0.001) but higher median white blood cell, platelet, and absolute lymphocyte counts (*p* = 0.014, < 0.001, and < 0.001, respectively) (Table 2). Peripheral smear was performed in 309 patients (45.7%), and Downey cells were observed in 131 (39.9%). The frequency of Downey cells did not differ between age groups (*p* = 0.543) (Table 1). Serum AST and ALT levels were available for 568 patients (81.7%). Median ALT levels were significantly higher in patients older than four years (*p* < 0.001), while median AST levels did not differ significantly (*p* = 0.63). No statistically significant differences were found between age groups in median ESR or CRP levels (*p* = 0.71 and 0.059, respectively) (Table 2).

**Figure 1:**
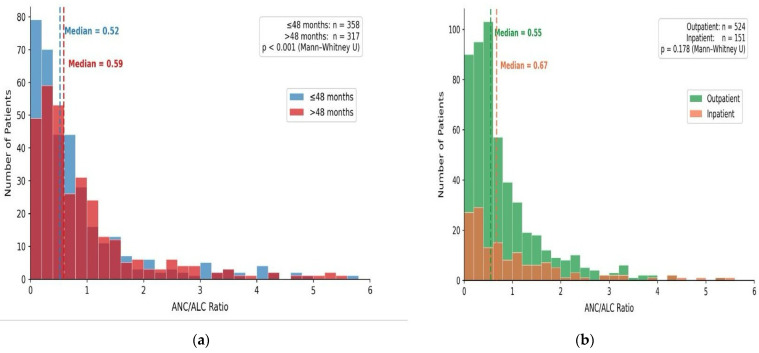
(**a**) Distribution of ANC/ALC ratio by age group. (**b**) Distribution of ANC/ALC ratio by hospitalization status. Abbreviations: ANC, absolute neutrophil count; ALC, absolute lymphocyte count.

The ANC/ALC ratio was significantly higher in children older than 48 months compared with younger children (median 0.64 vs. 0.52, *p* < 0.001), whereas no significant difference was observed in the AST/ALT ratio between age groups. When compared by hospitalization status, the ANC/ALC ratio showed a non-significant trend toward higher values in hospitalized patients (median 0.69 vs. 0.54, *p* = 0.178); the AST/ALT ratio did not differ significantly between groups. The distribution of ANC/ALC ratios across age groups and hospitalization status is illustrated in Figure 1a,b.

Cervical ultrasonography was performed in 271 patients, all of whom had lymphadenopathy; submandibular in 243 (89.7%), cervical in 139 (54%), submental in 2 (0.7%), and supraclavicular in 1 (0.4%). Abdominal ultrasonography was performed in 274 patients, revealing organomegaly in 145 (52.9%), while findings were normal in 129 (47.1%). The frequency of submandibular and cervical lymphadenopathy did not differ significantly between age groups (*p* = 0.49 and 0.29, respectively); however, organomegaly and splenomegaly were significantly more common among children older than four years (*p* = 0.018 and < 0.001, respectively) (Table 1).

### 3.5. Multivariable Logistic Regression Analyses

To identify independent predictors of key clinical outcomes, three separate multivariable binary logistic regression models were constructed. We performed three separate multivariable binary logistic regression models to examine independent predictors of: (1) hospitalization, (2) classic infectious mononucleosis (IM) phenotype, and (3) antibiotic use. Detailed regression outputs are provided in Supplementary Tables S1–S3.

#### 3.5.1. Hospitalization Model

The model was statistically significant (χ^2^ = 36.2, df = 23, *p* = 0.039). Among 23 independent variables entered, three were independently associated with hospitalization: lymphopenia (OR = 3.42, 95% CI: 1.28–9.17, *p* = 0.015), lymphadenopathy (OR = 1.60, 95% CI: 1.01–2.52, *p* = 0.043), and vomiting (OR = 1.80, 95% CI: 1.01–3.21, *p* = 0.048). Age group (≤4 vs. >4 years) was not independently associated with hospitalization after adjusting for these variables (OR = 0.90, 95% CI: 0.58–1.41, *p* = 0.651).

#### 3.5.2. Classic IM Phenotype Model

This model demonstrated excellent fit (χ^2^ = 167, df = 25, *p* < 0.001; McFadden R^2^ = 0.371). Independent predictors of the classic IM phenotype included lymphadenopathy (OR = 69.89, 95% CI: 8.95–545.97, *p* < 0.001), thrombocytosis (OR = 5.55, 95% CI: 2.02–15.26, *p* < 0.001), ear pain (OR = 6.36, 95% CI: 1.18–34.34, *p* = 0.032), risk score category 2 vs. 0 (OR = 5.50, 95% CI: 1.95–15.57, *p* = 0.001), risk score category 1 vs. 0 (OR = 4.38, 95% CI: 1.74–11.03, *p* = 0.002), and tonsillar hypertrophy (OR = 3.09, 95% CI: 1.29–7.39, *p* = 0.011). Age group did not reach independent significance in this model (OR = 1.43, 95% CI: 0.74–2.76, *p* = 0.293), suggesting that the age-related difference in classic IM presentation is largely mediated by these clinical and laboratory features.

#### 3.5.3. Antibiotic Use Model

The model was significant (χ^2^ = 70.1, df = 30, *p* < 0.001). Only two variables were independently associated with antibiotic use: rhinorrhea, which was inversely associated (OR = 0.64, 95% CI: 0.41–0.99, *p* = 0.045), and CRP level, which showed a marginal positive association (OR = 1.001, 95% CI: 1.000–1.001, *p* = 0.048). Age group, lymphadenopathy, tonsillitis, and other clinical features did not independently predict antibiotic use.

### 3.6. Diagnostic Performance of ANC/ALC and AST/ALT Ratios and Derivation of a Composite Risk Score

**Figure 2:**
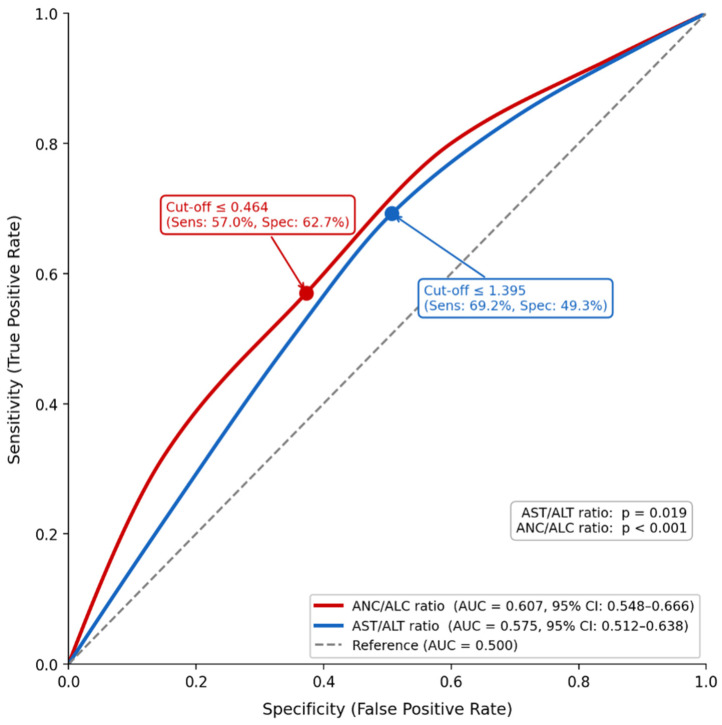
Receiver operating characteristic (ROC) curves for AST/ALT and ANC/ALC ratios in predicting the classic infectious mononucleosis phenotype. The classic infectious mononucleosis phenotype was defined as the concurrent presence of fever, pharyngitis, and lymphadenopathy. Abbreviations: AUC = area under the curve; CI = confidence interval; AST = aspartate aminotransferase; ALT = alanine aminotransferase; ANC = absolute neutrophil count; ALC = absolute lymphocyte count.

ROC curve analysis demonstrated modest but statistically significant discriminative ability for both the ANC/ALC ratio (AUC = 0.607, 95% CI: 0.548–0.666, *p* < 0.001) and the AST/ALT ratio (AUC = 0.575, 95% CI: 0.512–0.638, *p* = 0.019) in identifying the classic IM phenotype (Figure 2). The optimal cut-off values were ≤0.464 for ANC/ALC and ≤1.395 for AST/ALT, with negative predictive values exceeding 90% for both markers (Figure 2).

Based on these thresholds, a composite risk score (range 0–2) was derived. In multivariable logistic regression adjusted for age group, the model was statistically significant (χ^2^ = 23.3, df = 3, *p* < 0.001). Compared with a score of 0, a score of 1 was associated with increased odds of classic IM presentation (OR = 1.99, 95% CI: 1.01–3.94, *p* = 0.047), and a score of 2 with substantially higher odds (OR = 3.47, 95% CI: 1.73–6.96, *p* < 0.001). Age >48 months also remained an independent predictor (OR = 1.94, 95% CI: 1.17–3.23, *p* = 0.011).

Estimated marginal probabilities increased progressively with higher risk scores and older age (Table 3), ranging from 5.4% to 16.4% in children ≤48 months and from 9.9% to 27.6% in those >48 months.

### 3.7. Treatment and Follow-Up

Of the total patients, 544 (78.3%) were managed on an outpatient basis, while 151 (21.7%) required hospitalization. The median age of hospitalized patients was 3.8 years (IQR: 1.9–6.6 years). There was no significant difference in hospitalization rates between age groups (*p* = 0.923) (Table 1). Among hospitalized patients, fever, rhinorrhea, and vomiting were significantly more frequent (*p* = 0.003, 0.031, and 0.005, respectively), and physical examination more often revealed tonsillar hypertrophy, tonsillitis, lymphadenopathy, and organomegaly (*p* = 0.01, <0.001, 0.017, and 0.003, respectively) (Table 4). Additionally, hospitalized patients had significantly higher WBC, ANC, ESR, and CRP levels compared with outpatients (*p* = 0.001, 0.002, 0.001, and 0.001, respectively) (Table 5).

The median duration of hospital stay was 5 days (IQR: 4–7), with no significant age-related difference (*p* = 0.43). Empirical antibiotic therapy was initiated in 446 patients (64.2%), with no significant association between antibiotic use and age group (*p* = 0.137). Throat cultures were obtained from 268 patients (38.6%), and GABHS was isolated in 20 cases (7.5%), for which appropriate antibiotics were administered. Post-infection follow-up was completed in 440 patients (66.3%) in the outpatient setting. The median time to clinical recovery was 13 days (IQR: 10–16), with no significant difference between age groups (*p* = 0.28).

## 4. Discussion

This study provides a comprehensive real-world characterization of primary EBV infection in a large pediatric cohort, including both inpatient and outpatient cases, a distinction that remains relatively underrepresented in the existing literature. Our findings demonstrate a clear age-dependent clinical spectrum: children aged ≤4 years are more commonly presenting with nonspecific respiratory and gastrointestinal symptoms, whereas older children are more frequently exhibiting the classic infectious mononucleosis phenotype. Importantly, multivariable analyses revealed that age itself was not an independent predictor of hospitalization, classic IM phenotype, or antibiotic use after adjustment for clinical and laboratory variables. Instead, specific findings—including lymphopenia, lymphadenopathy, vomiting, thrombocytosis, and tonsillar hypertrophy—emerged as the principal determinants of key clinical outcomes. In addition, a composite risk score derived from ANC/ALC and AST/ALT ratios demonstrated high negative predictive value, supporting its potential utility as a simple bedside rule-out tool for the classic IM phenotype. Collectively, these findings provide clinically relevant insight into the diagnostic heterogeneity of pediatric EBV infection and highlight the importance of integrating clinical and laboratory parameters into age-specific diagnostic and antimicrobial stewardship strategies.

EBV seroprevalence and the age at primary infection vary considerably by geographic region and socioeconomic conditions, occurring earlier in developing countries and later in industrialized settings [8,15,16,17]. Consistent with previous reports from our country, more than half of our patients were aged ≤4 years and approximately 90% were under 10 years old [11,18].

The clinical presentation of primary EBV infection is closely age-dependent. Nonspecific symptoms predominate in young children, while 30–45% of adolescents and young adults develop the classic IM syndrome of fever, tonsillopharyngitis, and lymphadenopathy [19,20,21,22]. Consistent with these reports, rash, diarrhea, and respiratory symptoms were more frequent in younger children in our cohort, whereas sore throat, dysphagia, and lymphadenopathy predominated in older age groups, with hepatosplenomegaly also observed more frequently in children older than four years. These patterns support the well-described age-related clinical transition of primary EBV infection. Importantly, however, multivariable logistic regression analyses revealed that age group was not an independent predictor of hospitalization, classic IM phenotype, or antibiotic use after adjustment for clinical and laboratory variables. Instead, specific findings—lymphopenia, lymphadenopathy, and vomiting for hospitalization, and lymphadenopathy, thrombocytosis, and tonsillar hypertrophy for the classic IM phenotype—emerged as the primary independent determinants of these outcomes. To further strengthen diagnostic discrimination, a composite risk score was derived from ROC-optimized cut-off values for ANC/ALC and AST/ALT ratios. Both markers demonstrated modest but statistically significant discriminative ability (AUC 0.607 and 0.575, respectively); however, given the low prevalence of the classic IM phenotype in our cohort, the composite score is best interpreted as a rule-out rather than a rule-in tool, a role supported by negative predictive values exceeding 90%. The score showed a stepwise increase in the probability of classic IM presentation across age groups and risk categories, providing a simple framework for clinical risk stratification pending external validation [12,13].

Rash occurs in approximately 15–34% of children with primary EBV infection and is more frequent in younger age groups [23]. In our cohort, rash frequency was consistent with prior reports and was significantly more common in children aged ≤4 years. Antibiotic-associated rash was observed in 51% of those who received antibiotics; however, the occurrence of rash in younger children without antibiotic exposure suggests that this association may partly reflect age-related differences in immune response rather than a purely drug-induced phenomenon. Lymphocytosis and atypical lymphocytes on peripheral smear are additional characteristic features of primary EBV infection, with atypical lymphocytosis reported more frequently in older children and elevated WBC, ALC, and neutropenia more common in younger children [17,23].

Although mild to moderate transient elevations in transaminase levels are frequently observed during primary EBV infection, clinically significant hepatitis and liver failure are uncommon. Hepatic involvement is a well-recognized feature of primary EBV infection, encompassing both biochemical abnormalities and structural changes detectable on ultrasonography [24,25]. Elevated transaminase levels were common in our cohort, consistent with the high prevalence of EBV-associated subclinical hepatitis in pediatric populations [23,25]. Hepatomegaly and splenomegaly were also frequently identified, underscoring the role of imaging in evaluation [25]. In line with prior studies, ALT elevation was more pronounced in older children, likely reflecting age-related differences in immune response [4,5]. From a clinical perspective, most enzyme elevations were mild and self-limiting; even marked ALT elevations may persist for weeks without indicating severe liver disease or requiring antiviral therapy [24,26]. These findings support routine liver function testing and follow-up monitoring, particularly in hospitalized patients and those with hepatomegaly [26].

The higher frequency of organomegaly on abdominal ultrasonography in older children further supports the age-related clinical transition of primary EBV infection [10]. Although a systematic review suggested that routine liver function testing and abdominal ultrasonography may not be necessary in all immunocompetent patients [25], the high prevalence of transaminase elevation and hepatosplenomegaly in our cohort—particularly in older children—supports their inclusion in the diagnostic workup. Younger children additionally demonstrated higher WBC, ALC, and platelet counts, whereas older children showed significantly higher hemoglobin and ALT levels, likely reflecting age-related differences in immune response. GABHS co-infection, which occurs in approximately 5% of primary EBV cases [12,13], and the recognition of viral tonsillopharyngitis through complete blood count findings and peripheral smear evaluation are important considerations for reducing unnecessary antibiotic use [26].

In our study, despite the high rate of antibiotic use, the proportion of patients who underwent throat culture was low, and a peripheral smear was not performed in most cases. During the study period, rapid streptococcal antigen testing was not routinely available, and throat cultures were performed in a limited number of patients. In addition, the small number of GABHS-positive cases precluded a reliable statistical analysis to distinguish bacterial co-infection from primary EBV infection. Therefore, our findings regarding antibiotic use should be interpreted with caution in the absence of systematic microbiological confirmation. Taken together, these findings highlight an important gap in diagnostic practice. To reduce unnecessary antibiotic use, we recommend the routine implementation of peripheral smear evaluation for atypical lymphocytes, rapid streptococcal antigen testing, and throat culture as part of a standardized diagnostic approach. Adoption of this integrated strategy would not only improve diagnostic accuracy but also promote rational antibiotic use and contribute to efforts to limit antimicrobial resistance.

Although primary EBV infection is generally self-limiting, complications may occur in a minority of cases. Most existing studies are limited to hospitalized patients [9,10,12,13], which may lead to underestimation of the true clinical variability and disease burden, particularly in young outpatient children. By including both inpatient and outpatient cases, our study addresses this gap and provides a more representative characterization of primary EBV infection across the pediatric age spectrum. Hospitalized patients more frequently presented with fever, rhinorrhea, and vomiting, and showed higher rates of tonsillar hypertrophy, tonsillitis, lymphadenopathy, and organomegaly on physical examination, alongside significantly elevated WBC, ANC, CRP, and ESR levels—consistent with a more pronounced systemic inflammatory response. Although recovery time appeared longer in outpatients, this should not be interpreted as a true difference in disease course, as it is likely attributable to measurement bias and heterogeneity in follow-up practices.

This study has several important strengths. It includes one of the largest single-center pediatric cohorts of primary EBV infection reported to date, encompassing 695 patients across a broad age spectrum and including both inpatient and outpatient cases, thereby providing a more representative characterization of the clinical spectrum of pediatric EBV infection. Beyond descriptive analyses, multivariable logistic regression models were used to identify independent predictors of hospitalization, classic IM phenotype, and antibiotic use, offering clinically actionable insights. In addition, a composite risk score derived from ROC-optimized ANC/ALC and AST/ALT ratios demonstrated high negative predictive values, supporting its potential utility as a simple bedside rule-out tool. Several limitations should nonetheless be acknowledged. The retrospective design restricted the availability of detailed data on symptom onset, clinical progression, and the temporal relationships between EBV diagnosis, GABHS detection, and antibiotic initiation. Patients were not assessed under a standardized protocol, and limited microbiological data reduced the ability to reliably differentiate bacterial co-infection from primary EBV infection. The study period (2010–2015) may affect contemporary generalizability in light of evolving EBV epidemiology, although the observed clinical patterns and diagnostic challenges remain relevant. Further age stratification was not performed due to limited subgroup sizes, which may reduce statistical power. The composite risk score was internally derived and tested within the same dataset, limiting generalizability and necessitating external validation before clinical application. Data on disease severity and complications were not systematically recorded and may be underrepresented in predominantly outpatient cohorts. Finally, the absence of long-term follow-up data precluded a comprehensive assessment of prognosis. Future prospective multicenter studies with longitudinal follow-up are therefore warranted to clarify the clinical course and long-term outcomes of pediatric EBV infection.

## 5. Conclusions

This large retrospective study provides a comprehensive real-world characterization of primary EBV infection in children across age groups and clinical settings. Our findings demonstrate a clear age-related clinical spectrum, with younger children presenting with atypical features and older children more frequently exhibiting the classic infectious mononucleosis phenotype. However, multivariable analyses indicate that clinical and laboratory findings—rather than age alone—are the primary determinants of key outcomes. A simple composite risk score based on ANC/ALC and AST/ALT ratios showed modest discriminative ability but may aid in clinical risk stratification, particularly as a rule-out tool. In addition, the high rate of empirical antibiotic use highlights the need for improved diagnostic strategies and antimicrobial stewardship.

These findings support the development of age-specific diagnostic approaches and underscore the need for prospective multicenter studies to validate these tools and further define clinical outcomes in pediatric EBV infection.

## Figures and Tables

**Table 1:** Comparison of clinical, laboratory, imaging, and treatment characteristics according to age groups.

	Total	≤4 Years	>4 Years	
		*n* (%)	*n* (%)	*p* *
**Presenting symptoms**	695	372	323	
Fever	421 (60.6)	231 (62.1)	190 (58.8)	0.406
Sore throat	336 (48.3)	142 (38.2)	194 (60.1)	**<0.001**
Cervical swelling	265 (38.1)	126 (33.9)	139 (43)	0.111
Rhinorrhea	217 (31.7)	135 (36.3)	82 (25.4)	**0.002**
Cough	192 (27.8)	126 (33.9)	67 (20.7)	**<0.001**
Rash	186 (26.8)	124 (33.3)	62 (19.2)	**<0.001**
Loss of appetite	134 (19.3)	60 (16.1)	74 (22.9)	**0.022**
Dysphagia	105 (15.1)	41 (11)	64 (19.8)	**<0.001**
Vomiting	85 (12.7)	51 (13.7)	37 (11.5)	0.388
Diarrhea	55 (7.9)	44 (11.8)	11 (3.4)	**<0.001**
Eyelid edema	54 (7.8)	31 (8.3)	23 (7.1)	0.566
IM triad **	93 (13.3)	36 (9.7)	57 (17.6)	**<0.001**
**Physical examination findings**	695	372 (53.5)	323 (46.5)	
Tonsillar hypertrophy	459 (66)	241 (64.8)	218 (67.5)	0.319
Lymphadenopathy	364 (52.4)	139 (37.4)	225 (69.7)	**<0.001**
Tonsillitis	270 (38.8)	133 (35.8)	137 (42.4)	0.063
Rash	195 (28.1)	132 (35.5)	63 (19.5)	**<0.001**
Fever	172 (24.7)	103 (27.7)	69 (21.4)	0.06
Postnasal drip	85 (12.2)	47 (12.6)	38 (11.8)	0.748
Periorbital edema	56 (8.1)	26 (7)	30 (9.3)	0.748
Hepatomegaly	54 (7.8)	33 (8.9)	21 (6.5)	0.253
Hepatosplenomegaly	47 (6.8)	15 (4)	32 (9.9)	**0.002**
Splenomegaly	34 (4.9)	15 (4)	19 (5.9)	0.253
**Findings on peripheral blood smear**	309	162	147	
Downey cells	131 (39.9)	67 (41.4)	64 (43.5)	0.543
**Ultrasonographic findings**				
***Cervical ultrasonography***	271	133	138	
Cervical lymphadenopathy	123 (45.4)	56 (42.1)	67 (48.6)	0.287
***Abdominal ultrasonography***	274	138	137	
Hepatomegaly	84 (30.7)	37 (26.8)	47 (34.3)	0.117
Splenomegaly	127 (46.4)	50 (36.2)	77 (56.2)	**<0.001**
Organomegaly	145 (52.9)	63 (45.7)	82 (59.9)	**0.018**
**Treatment**				
Antibiotic therapy	446 (64.2)	230 (61.7)	216 (67.1)	0.137
Antibiotic-associated rash	100 (22.4)	67 (29.1)	33 (15.3)	**<0.001**
**Follow-up**	695	363	332	
Inpatient	151 (21.7)	81 (22.3)	70 (21.1)	0.923
Outpatient	544 (78.3)	282 (77.7)	262 (78.9)	

* Chi-square test (χ^2^), ** IM (Infectious mononucleosis) triad: fever, sore throat, cervical swelling. Statistically significant results are indicated in bold.

**Table 2:** Comparison of laboratory parameters and clinical follow-up according to age groups.

	All Patients (*n* = 695)		≤4 Years (*n* = 372)		>4 Years (*n* = 323)			
	Patients Tested *n* (%)	Median (IQR)	Patients Tested (%)	Median (IQR)	Patients Tested (%)	Median (IQR)	Test Statistic	*p* *
**Laboratory findings**								
Hgb (g/dL)	675 (97.1)	12 (11.3–12.8)	358 (96.2)	11.6 (11–12.3)	317 (98.1)	12.5 (11.9–13.1)	82.75	**<0.001**
WBC (×10^3^/µL)	675 (97.1)	11.9 (8.7–15.7)	358 (96.2)	12.4 (9.1–16.1)	317 (98.1)	11.3 (8.4–15.1)	50.52	**0.014**
PLT (×10^3^/µL)	675 (97.1)	267 (201–344)	358 (96.2)	285.5 (207–360)	317 (98.1)	248 (193.5–320)	47.17	**<0.001**
ANC (×10^3^/µL)	675 (97.1)	3.37 (2.3–5.1)	358 (96.2)	3.3 (2.3–5.1)	317 (98.1)	3.5 (2.3–5.1)	58.97	0.38
ALC (×10^3^/µL)	675 (97.1)	5.7 (3.64–8.2)	358 (96.2)	6.2 (4.3–8.9)	317 (98.1)	5.1 (3.1–7.7)	45.27	**<0.001**
AST (U/L)	568 (81.7)	45 (31–75)	291 (78.2)	44 (32–68)	277 (85.8)	47 (28.5–88)	41.24	0.63
ALT (U/L)	568 (81.7)	33 (18–93)	291 (78.2)	27 (17–65)	277 (85.8)	44 (20–116.5)	47.6	**<0.001**
ESR (mm/h)	443 (63.7)	22 (14–40)	229 (61.6)	23 (14–41.5)	214 (66.3)	22 (12–40)	23.9	0.71
CRP (mg/dL)	565 (81.3)	9 (3.6–22)	301 (80.9)	8 (3.4–20.5)	264 (81.8)	10.5 (4.6–25)	43.37	0.059
**Time to recovery (days)**	440 (63.6)	13 (10–16)	237 (63.7)	12 (10–16)	203 (62.8)	13 (10–17)	25.5	0.28
**Hospital stay (days)**	151 (21.7)	5 (4–7)	81 (21.8)	5 (4–7)	70 (21.7)	5 (4–7)	3.4	0.43

Abbreviations: Hgb, hemoglobin; WBC, white blood cell; PLT, platelet count; ANC, absolute neutrophil count; ALC, absolute lymphocyte count; AST, aspartate aminotransferase; ALT, alanine aminotransferase; ESR, erythrocyte sedimentation rate; CRP, C-reactive protein. * Mann–Whitney U test. Data are presented as median (interquartile range, 25th–75th percentile). Statistically significant results are indicated in bold.

**Table 3:** Estimated marginal probabilities of classic infectious mononucleosis presentation by composite risk score and age group.

Risk Score	≤48 Months Probability	≤48 Months 95% CI	>48 Months Probability	>48 Months 95% CI
0—Both ratios above cut-off	5.4%	3.0–9.5%	9.9%	5.6–17.0%
1—One ratio at/below cut-off	10.1%	6.4–15.7%	18.0%	12.7–24.8%
2—Both ratios at/below cut-off	16.4%	10.5–24.8%	27.6%	19.8–37.1%

**Note**. Probabilities were estimated from a multivariable logistic regression model incorporating the composite risk score and age group (χ^2^ = 23.3, df = 3, *p* < 0.001). The composite risk score ranges from 0 (lowest risk: both ANC/ALC and AST/ALT ratios above cut-off) to 2 (highest risk: both ratios at or below cut-off), based on ROC-derived cut-off values: ANC/ALC ratio ≤ 0.464 and AST/ALT ratio ≤ 1.395. Values represent the predicted probability of classic infectious mononucleosis presentation with 95% confidence interval. Abbreviations: ANC—absolute neutrophil count; ALC—absolute lymphocyte count; AST—aspartate aminotransferase; ALT—alanine aminotransferase.

**Table 4:** Comparison of clinical findings according to hospitalization status.

	Total	Outpatient	Inpatient	
	*n* (%)	*n* (%)	*n* (%)	*p* *
**Presenting symptoms**	695	544	151	
Fever	421 (60.6)	314 (57.5)	107 (70.9)	**0.003**
Sore throat	336 (48.3)	262 (48.2)	74 (49)	0.854
Cervical swelling	265 (38.1)	204 (37.5)	61 (40.4)	0.52
Rhinorrhea	217 (31.7)	159 (29.2)	58 (38.4)	**0.031**
Cough	192 (27.8)	147 (27)	45 (29.8)	0.499
Rash	186 (26.8)	149 (27.4)	37 (24.5)	0.478
Loss of appetite	134 (19.3)	97 (17.8)	37 (24.5)	0.066
Dysphagia	105 (15.1)	81 (14.9)	24 (15.9)	0.86
Vomiting	85 (12.7)	56 (10.3)	29 (19.2)	**0.005**
Diarrhea	55 (7.9)	45 (8.3)	10 (6.6)	0.621
Eyelid edema	54 (7.8)	41 (7.5)	13 (8.6)	0.792
IM triad **	93 (13.3)	69 (12.7)	24 (15.9)	0.373
**Physical examination findings**	695	544	151	
Tonsillar hypertrophy	459 (66)	364 (63.6)	113 (74.8)	**0.01**
Lymphadenopathy	364 (52.4)	272 (50)	92 (60.9)	**0.017**
Tonsillitis	270 (38.8)	191 (35.1)	79 (52.3)	**<0.001**
Rash	195 (28.1)	153 (28.1)	42 (27.8)	0.94
Fever	172 (24.7)	130 (23.9)	42 (27.8)	0.324
Postnasal drip	85 (12.2)	68 812.5)	17 (11.3)	0.786
Periorbital edema	56 (8.1)	46 (8.5)	10 (6.6)	0.573
Hepatomegaly	54 (7.8)	37 (6.8)	17 (11.3)	0.101
Hepatosplenomegaly	47 (6.8)	32 (5.9)	15 (9.9)	0.116
Splenomegaly	34 (4.9)	24 (4.4)	10 (6.6)	0.368
Organomegaly	135 (19.4)	93 (17.1)	42 (27.8)	**0.003**

* Chi-square test (χ^2^), ** IM triad: fever, sore throat, cervical swelling. Abbreviations: IM—infectious mononucleosis. Statistically significant results are indicated in bold.

**Table 5:** Comparison of laboratory findings and recovery times according to hospitalization status.

	Outpatient (*n* = 544)		Inpatient (*n* = 151)			
	Patients Tested. *n* (%)	Median (IQR)	Patients Tested. *n* (%)	Median (IQR)	Test Statistic	*p* *
**Laboratory findings**						
Hgb (g/dL)	524 (96.3)	12.1 (11.4–12.8)	151 (100)	12 (11.1–13)	37.62	0.357
WBC (×10^3^/µL)	524 (96.3)	11.5 (8.6–15.3)	151 (100)	13.4 (9.7–18.1)	46.49	**0.001**
Platelet count (×10^3^/µL)	524 (96.3)	270 (205–344)	151 (100)	242 (185–338)	35.46	0.052
ANC (×10^3^/µL)	524 (96.3)	3.3 (2.3–4.9)	151 (100)	3.8 (2.7–6.3)	46.02	**0.002**
ALC (×10^3^/µL)	524 (96.3)	5.6 (3.6–8.0)	151 (100)	6.1 (3.6–9.2)	41.8	0.288
AST (U/L)	420 (77.2)	43 (31–74)	148 (98)	48.5 (33.3–84.8)	33.81	0.112
ALT (U/L)	419 (77)	32 (18–88)	148 (98)	35.5 (17.5–101.8)	32.25	0.468
ESR (mm/saat)	315 (57.9)	21 (12–38)	128 (84.8)	28 (17–45)	24.3	**0.001**
CRP (mg/dL)	417 (76.7)	8 (3.4–19.7)	148 (98)	12 (6–31)	36.75	**0.001**
**Time to recovery (days)**	313 (57.5)	14 (10–17)	127 (84.1)	11 (9–14)	15.39	**<0.001**

Abbreviations: Hgb, hemoglobin; WBC, white blood cell; PLT, platelet count; ANC, absolute neutrophil count; ALC, absolute lymphocyte count; AST, aspartate aminotransferase; ALT, alanine aminotransferase; ESR, erythrocyte sedimentation rate; CRP, C-reactive protein. * Mann–Whitney U test. Data are presented as median (interquartile range, 25th–75th percentile). Statistically significant results are indicated in bold.

## Data Availability

The datasets generated and/or analyzed during the current study are not publicly available but are available from the corresponding author on reasonable request.

## Supplementary Materials

The following supporting information can be downloaded at: https://www.mdpi.com/article/10.3390/pathogens15050554/s1. Table S1: Multivariable logistic regression analysis for predictors of hospitalization; Table S2: Multivariable logistic regression analysis for predictors of the classic infectious mononucleosis phenotype; Table S3: Multivariable logistic regression analysis for predictors of antibiotic use.

## Abbreviations

ALC	Absolute lymphocyte count
ALT	Alanine aminotransferase
ANC	Absolute neutrophil count
AST	Aspartate aminotransferase
AUC	Area under the curve
CI	Confidence interval
CRP	C-reactive protein
EBNA	Epstein–Barr nuclear antigen
EBV	Epstein–Barr virus;
ESR	Erythrocyte sedimentation rate
GABHS	Group A ß-Hemolitic *Streptococ*
Hgb	Hemoglobin
IM	Infectious mononucleosis
IQR	Interquartile range
NPV	Negative predictive value
OR	Odds ratio
PLT	Platelet count
PPV	Positive predictive value
ROC	Receiver operating characteristic
SD	Standard deviation
VCA	Viral capsid antigen
WBC	White blood cell
