# Supplementary material for: Age-Dependent Clinical Patterns of Primary Epstein–Barr Virus Infection in Children: Insights for Diagnostic Accuracy
Source: Pathogens. 2026 May 20;15(5):554. doi: 10.3390/pathogens15050554 (PMC13209608; doi:10.3390/pathogens15050554)
Supplement: Supplementary file 1 [file pathogens-15-00554-s001.zip › pathogens-4278182-supplementary.pdf]

Supplementary Table S1. Multivariable logistic regression analysis for predictors of hospitalization.

Predictor	Estimate	SE	Z	*p*	OR	95% CI Lower	95% CI Upper
Intercept	−2.174	0.383	−5.677	<.001	0.114	0.054	0.241
Age group (>48 months vs. ≤48 months)	−0.102	0.225	−0.452	0.651	0.903	0.581	1.405
WBC	0.000	0.000	1.604	0.109	1.000	1.000	1.000
Fever	0.381	0.225	1.689	0.091	1.464	0.941	2.277
Sore throat	−0.052	0.234	−0.220	0.826	0.950	0.601	1.501
Cough	−0.062	0.238	−0.262	0.793	0.940	0.589	1.498
Rash	0.346	0.259	1.337	0.181	1.413	0.851	2.347
Rhinorrhea	0.227	0.230	0.988	0.323	1.255	0.800	1.968
Poor appetite	0.116	0.249	0.464	0.643	1.122	0.689	1.828
Abdominal pain	−0.374	0.381	−0.984	0.325	0.688	0.326	1.450
Dysphagia	−0.176	0.302	−0.584	0.559	0.838	0.464	1.514
**Vomiting**	**0.586**	**0.296**	**1.982**	**0.048**	**1.796**	**1.007**	**3.206**
Diarrhea	−0.423	0.412	−1.027	0.304	0.655	0.292	1.468
Ear pain	0.181	0.725	0.250	0.803	1.199	0.289	4.967
Eyelid edema	0.560	0.503	1.113	0.266	1.751	0.653	4.698
**Lymphadenopathy**	**0.469**	**0.232**	**2.023**	**0.043**	**1.599**	**1.015**	**2.520**
GABHS positivity	0.517	0.576	0.897	0.370	1.677	0.542	5.185
Periorbital edema	−0.912	0.534	−1.709	0.088	0.402	0.141	1.144
Abdominal tenderness	0.480	0.869	0.552	0.581	1.615	0.294	8.876
Thrombocytopenia	0.177	0.304	0.581	0.561	1.193	0.657	2.165
**Lymphopenia**	**1.229**	**0.503**	**2.443**	**0.015**	**3.419**	**1.275**	**9.165**
Lymphocytosis	0.087	0.283	0.307	0.759	1.091	0.627	1.898
Risk score (ref: 0)							
Score 1 vs. 0	−0.059	0.246	−0.240	0.810	0.943	0.582	1.526
Score 2 vs. 0	0.082	0.313	0.262	0.793	1.085	0.588	2.004

Note. Bold rows indicate statistically significant predictors ($p < 0.05$). OR = odds ratio; CI = confidence interval; SE = standard error; WBC = white blood cell count; GABHS = Group A β-hemolytic Streptococcus. The model included 23 independent variables ($\chi^2 = 36.2$, df = 23, $p = 0.039$). Reference category for the dependent variable: not hospitalized.

Supplementary Table S2. Multivariable logistic regression analysis for predictors of the classic infectious mononucleosis phenotype.

Predictor	Estimate	SE	Z	*p*	OR	95% CI Lower	95% CI Upper
Intercept	−7.591	1.233	−6.157	<.001	0.001	0.000	0.006
Age group (>48 months vs. ≤48 months)	0.354	0.337	1.052	0.293	1.425	0.737	2.756
Cough	−0.266	0.369	−0.720	0.471	0.767	0.372	1.580
Rash (symptom)	−0.933	1.061	−0.879	0.379	0.394	0.049	3.145
Rhinorrhea	−0.041	0.352	−0.117	0.907	0.960	0.482	1.912
Poor appetite	−0.282	0.376	−0.748	0.454	0.755	0.361	1.578
Abdominal pain	0.075	0.576	0.130	0.897	1.078	0.348	3.334
Dysphagia	0.259	0.396	0.653	0.514	1.295	0.596	2.812
Vomiting	−0.805	0.550	−1.463	0.143	0.447	0.152	1.314
Diarrhea	−0.379	0.736	−0.515	0.607	0.685	0.162	2.899
**Ear pain**	**1.850**	**0.861**	**2.149**	**0.032**	**6.357**	**1.177**	**34.341**
Eyelid edema	−1.195	0.989	−1.209	0.227	0.303	0.044	2.102
**Lymphadenopathy**	**4.247**	**1.049**	**4.049**	**<.001**	**69.893**	**8.947**	**545.967**
Periorbital edema	−0.860	0.856	−1.004	0.315	0.423	0.079	2.267
Thrombocytopenia	0.325	0.447	0.727	0.467	1.384	0.577	3.321
Lymphopenia	−0.420	1.216	−0.345	0.730	0.657	0.061	7.127
Lymphocytosis	−0.026	0.361	−0.072	0.943	0.975	0.481	1.975
**Thrombocytosis**	**1.714**	**0.516**	**3.323**	**<.001**	**5.552**	**2.020**	**15.261**
Neutropenia	−0.913	0.718	−1.272	0.204	0.401	0.098	1.640
Tonsillitis	0.467	0.340	1.373	0.170	1.594	0.819	3.103
**Tonsillar hypertrophy**	**1.128**	**0.445**	**2.534**	**0.011**	**3.089**	**1.291**	**7.392**
Hepatosplenomegaly	0.147	0.534	0.275	0.784	1.158	0.406	3.299
Rash (physical exam)	−1.769	1.056	−1.675	0.094	0.171	0.022	1.351
Risk score (ref: 0)							
**Score 1 vs. 0**	**1.477**	**0.471**	**3.137**	**0.002**	**4.382**	**1.741**	**11.027**
**Score 2 vs. 0**	**1.705**	**0.531**	**3.214**	**0.001**	**5.503**	**1.946**	**15.568**

Note. Bold rows indicate statistically significant predictors ($p < 0.05$). OR = odds ratio; CI = confidence interval; SE = standard error. The model included 25 independent variables and demonstrated excellent fit ($\chi^2 = 167$, df = 25, $p < 0.001$; McFadden $R^2 = 0.371$; AIC = 335). Reference category for the dependent variable: absence of classic IM phenotype. Risk score was derived from ANC/ALC and AST/ALT ratios using ROC-defined cut-off values.

Supplementary Table S3. Multivariable logistic regression analysis for predictors of antibiotic use.

Predictor	Estimate	SE	Z	*p*	OR	95% CI Lower	95% CI Upper
Intercept	0.848	0.427	1.988	0.047	2.334	1.012	5.386
Age group (>48 months vs. ≤48 months)	0.293	0.216	1.352	0.176	1.340	0.877	2.047
Cough	0.320	0.238	1.346	0.178	1.377	0.864	2.194
Rash (symptom)	−0.482	0.431	−1.117	0.264	0.618	0.266	1.438
**Rhinorrhea**	**−0.452**	**0.225**	**−2.006**	**0.045**	**0.636**	**0.409**	**0.990**
Poor appetite	−0.438	0.235	−1.861	0.063	0.645	0.407	1.023
Abdominal pain	−0.397	0.334	−1.189	0.235	0.672	0.349	1.294
Dysphagia	−0.420	0.283	−1.483	0.138	0.657	0.377	1.144
Vomiting	−0.034	0.295	−0.114	0.909	0.967	0.542	1.724
Diarrhea	0.200	0.372	0.537	0.591	1.221	0.589	2.529
Ear pain	−0.167	0.690	−0.242	0.809	0.846	0.219	3.270
Eyelid edema	−0.238	0.474	−0.502	0.616	0.788	0.311	1.997
Lymphadenopathy	0.183	0.219	0.836	0.403	1.201	0.781	1.847
Periorbital edema	−0.583	0.470	−1.240	0.215	0.558	0.222	1.403
Abdominal tenderness	−1.389	0.786	−1.768	0.077	0.249	0.054	1.163
Thrombocytopenia	−0.282	0.306	−0.920	0.357	0.755	0.414	1.374
Lymphopenia	1.111	0.713	1.560	0.119	3.038	0.752	12.279
Lymphocytosis	0.112	0.269	0.415	0.678	1.118	0.660	1.896
Thrombocytosis	−0.516	0.302	−1.711	0.087	0.597	0.331	1.078
Neutropenia	−0.382	0.391	−0.978	0.328	0.682	0.317	1.467
Tonsillitis	−0.129	0.229	−0.565	0.572	0.879	0.561	1.376
Tonsillar hypertrophy	0.323	0.239	1.353	0.176	1.381	0.865	2.205
Hepatosplenomegaly	−0.539	0.341	−1.579	0.114	0.584	0.299	1.139
Rash (physical exam)	−0.552	0.428	−1.290	0.197	0.576	0.249	1.332
WBC	0.000	0.000	−1.091	0.275	1.000	1.000	1.000
**CRP**	**0.001**	**0.000**	**1.981**	**0.048**	**1.001**	**1.000**	**1.001**
GABHS positivity	1.314	0.796	1.650	0.099	3.721	0.782	17.711
AST/ALT ratio	0.166	0.142	1.169	0.243	1.180	0.894	1.559
ANC/ALC ratio	0.039	0.065	0.598	0.550	1.040	0.916	1.180
Fever (physical exam)	−0.034	0.234	−0.145	0.885	0.967	0.611	1.529
Hepatomegaly	−0.070	0.335	−0.210	0.834	0.932	0.483	1.798

Note. Bold rows indicate statistically significant predictors ($p < 0.05$). OR = odds ratio; CI = confidence interval; SE = standard error; WBC = white blood cell count; CRP = C-reactive protein; AST = aspartate aminotransferase; ALT = alanine aminotransferase; ANC = absolute neutrophil count; ALC = absolute lymphocyte count; GABHS = Group A β-hemolytic Streptococcus. The model included 30 independent variables ($\chi^2 = 70.1$, df = 30, $p < 0.001$). Reference category for the dependent variable: no antibiotic use.
